# Multimodal Detection for Cryptogenic Epileptic Seizures Based on Combined Micro Sensors

**DOI:** 10.1155/2020/5734932

**Published:** 2020-09-07

**Authors:** Zhitian Shen, Yang Jiao, Yiwen Xu, Wei Shi, Chen Yang, Dan Li, Hongtao Ma, Weiwei Shao, Zhangjian Li, Yaoyao Cui

**Affiliations:** ^1^University of Science and Technology of China, 230026, China; ^2^Medical Acoustic Department, Suzhou Institute of Biomedical Engineering and Technology, Chinese Academy of Sciences, 215163, China; ^3^University of Chinese Academy of Sciences, 100049, China; ^4^Department of Radiology, The First Hospital of Jilin University, 130021, China; ^5^Department of Neurological Surgery, Weill Medical College of Cornell University, NY 10065, USA

## Abstract

The cryptogenic epilepsy of the neocortex is a disease in which the seizure is accompanied by intense cerebral nerve electrical activities but the lesions are not observed. It is difficult to locate disease foci. Electrocorticography (ECoG) is one of the gold standards in seizure focus localization. This method detects electrical signals, and its limitations are inadequate resolution which is only 10 mm and lack of depth information. In order to solve these problems, our new method with implantable micro ultrasound transducer (MUT) and photoplethysmogram (PPG) device detects blood changes to achieve higher resolution and provide depth information. The basis of this method is the neurovascular coupling mechanism, which shows that intense neural activity leads to sufficient cerebral blood volume (CBV). The neurovascular coupling mechanism established the relationship between epileptic electrical signals and CBV. The existence of mechanism enables us to apply our new methods on the basis of ECoG. Phantom experiments and in vivo experiments were designed to verify the proposed method. The first phantom experiments designed a phantom with two channels at different depths, and the MUT was used to detect the depth where the blood concentration changed. The results showed that the MUT detected the blood concentration change at the depth of 12 mm, which is the position of the second channel. In the second phantom experiments where a PPG device and MUT were used to monitor the change of blood concentration in a thick tube, the results showed that the trend of superficial blood concentration change provided by the PPG device is the same as that provided by the MUT within the depth of 2.5 mm. Finally, in the verification of in vivo experiments, the blood concentration changes on the surface recorded by the PPG device and the changes at a certain depth recorded by the MUT all matched the seizure status shown by ECoG. These results confirmed the effectiveness of the combined micro sensors.

## 1. Introduction

The approaches that detect the abnormal brain activities are now performing a variety of tasks in many fields. Computed tomography and magnetic resonance angiography can do some morphologic analysis, while inapparent morphological lesions still arouse in some diseases. In terms of the assessment of vascular hemodynamics, these methods have some limitations, especially for the CBV. So, it is essential to locate the lesions by detecting other relevant parameters.

Epilepsy is a sort of typical brain disease which is still one of the most difficult clinical problems in the world. It often resulted from brain dysfunction which is a sudden abnormal discharge of neuron. Seizures often occur in patients at frequencies varying from less than once a year to several times a day, which could have a huge impact on the patient's quality of life [1, 2]. For patients, invasive surgeries are needed to remove foci for treating their symptoms if seizures become drug-refractory and other necessary noninvasive medical options have failed. In the case of seizure, epilepsy may be induced or transmitted by one or more nonobvious lesions, in addition to obvious morphological abnormalities. So, to find and remove the hidden lesions are of great importance for curing disease. For surgeons to identify foci in surgical treatment, a reliable method is to map neural activity on the surface of the brain and localize the foci. The gold standard in seizure focus localization is intracranial implantation of an electrode, such as electrocorticography (ECoG) or stereoelectroencephalography (SEEG). Among them, ECoG detects the electrical signals on the surface of the brain to identify the lesion locations [3–5]. Because the resolution of ECoG is only 10 mm, removing some areas during surgery is often accompanied by excessive excision leading to surgical failure. Thus, other techniques are needed to improve resolution. Functional brain imaging techniques, such as fMRI, can offer high-resolution imaging results but cannot be used for seizure mapping because of the uncontrollable body movements. Besides, seizures are unpredictable and need a long time to observe and record. It is unrealistic to expose patients to fMRI for a long period of time. In order to solve these problems, researchers are urgently seeking a method and technology that can accurately locate the epileptic foci.

There is a neurovascular coupling mechanism in the human brain [6–8]. It illustrates that the changes in blood supply within the brain are consistent with the changes in brain activity [9, 10]. The CBV increases when the neuroactivity is active, and the CBV decreases when the neuroactivity is calm [11]. It means the change of the neuroactivity could be reflected by the hemodynamic changes of the corresponding area. Thus, the changes of blood in the brain can be detected to locate epileptic foci. One possible solution is photoplethysmography (PPG), which is a method for measuring the amount of light that is absorbed or reflected by blood vessels in living tissue [12]. Since the amount of optical absorption or reflection depends on the amount of blood that is present in the optical path, the PPG signal is responsive to changes in the volume of the blood [12]. It is usually used for monitoring blood pressure, measuring blood oxygen saturation, and recording changes in skin and muscle blood perfusion [12–14]. With the development of digital sensor and signal processing, PPG technology is becoming more readily available, inexpensive, convenient, and easily integrated into portable devices [15]. Different PPG devices use different wavelengths of light. Each light penetrates human tissue differently. Green and red infrared lights are often used, and the penetration depth is less than 1 mm and about 2.5 mm, respectively [14, 16, 17]. In studies of cortical cryptogenic epilepsy, some researchers have studied a LED-based optical device suitable for implantation to detect blood volume changes in the brain associated with epilepsy [18, 19]. It was combined with ECoG to locate lesions and verified its feasibility for in vivo experiments [18, 20, 21]. The size of this device is 2 mm × 2 mm × 0.8 mm. By using the suitable device and arrangement, this approach can further achieve a spatial resolution of 1 mm × 1 mm [22], which is far less than the ECoG resolution (10 mm) and suitable for implantation. Although this method has a great improvement in spatial resolution compared with ECoG, it can only recognize that if blood changes exist within the light detection depth and cannot provide a specific depth information. It still has some limitation for locating deep epileptic lesions deep in the brain.

In the field of ultrasound imaging, the method of detecting vascular hemodynamics is often relied on the Doppler method. Ultrasound has the ability to detect blood changes in depth. This can supplement the lack of depth information in PPG and ECoG. The parameters of blood flow within the vessels are shown by the Doppler spectrum of the target area. It could image the region of interest (ROI) and depict the real-time state of blood flow. Functional ultrasound (fUS) presented by Tanter and Fink is a suitable technique to image the cerebral blood volume [23–25]. This is capable of imaging the microvascular dynamics of the whole brain [26]. In their study, they advocated that the change of CBV at some areas of the brain has correspondence with the epileptic states [27]. Although the result in their study have shown that fUS has high temporal and spatial resolution for microvascular imaging, this method has the limitation in clinical application. In clinical practice, a few square centimeters of brain tissue is often examined. And the high-frequency ultrasound array probe used by fUS can only image and locate a section. This probe is too large to be implanted when the ECoG method is used at the same time. Besides, to detect cryptic epilepsy of the neocortex, the use pattern of the ECoG method usually requires continuous recording of brain activities which often last weeks or even months. So, it seems that the probe is not appropriate for clinical application. It is still challenging to find a suitable method to assist ECoG for the real-time observation of brain diseases. With the development of ultrasound in vivo, the size of high-frequency MUT can be as small as 0.5 mm × 0.5 mm. This type of transducer, such as intravascular ultrasound (Boston Scientific and Volcano) and endoscopic ultrasound microprobe (Olympus company and Fuji company), has been widely used in intravascular ultrasound. Therefore, miniaturized ultrasound transducers can be used to solve the problem of large probes for epilepsy detection.

In this paper, we proposed a novel method for locating epileptic lesions with MUT and PPG device. The MUT has a similar size with ECoG electrodes. This advantage determines that the MUT and PPG device could be used to be associated with ECoG electrodes to apply in clinical setting. In this approach, ECoG electrodes supply the precise time of the occurrence. The PPG device offer the precise position of the brain surface. The MUT complement the depth information of seizure. We tested and verified the ability of this method to detect blood concentration in phantom. We also measured the precise seizure site in in vivo rats by artificially inducing epilepsy. This method may have the prospect that the planted epilepsy 3D detection is feasible.

## 2. Materials and Methods

We designed phantom experiments and in vivo experiments, respectively, to verify the ability of MUT to detect blood changes and the feasibility of detecting by using PPG and MUT. For phantom experiments, we used PPG to record light signal. And we used MUT to record ultrasound signal for further analysis. For in vivo experiments, besides PPG and MUT, we added ECoG to record the local field potential (LFP) which is the electrical signal on the surface of the brain to reflect the brain activities for in vivo experiments. The phantom experiments used the same experiment setup and the data processing method with in vivo experiments.

### 2.1. Experiment Setup

ECoG electrodes were applied to record LFP. The voltage of LFP is about 1 mV, and it can easily be affected by noise. So, the AC Amplifier Model 1800 (A-M Systems, Sequim, WA) was used to amplify the electrical signal for 1000 times. The amplified signal was recorded by the data acquisition (DAQ) device USB-6008 (National Instruments, Austin, TX), whose sample rate is 1 kHz.

We used LED to emit light, whose wavelength is 530 nm, to the surface of the brain and used photodiode to record the intensity of the reflected light. The different concentrations of blood on the surface of the brain will reflect different intensity of light. This can be used to position the area of the surface precisely. The signals which photodiodes received were also amplified and recorded simultaneously with electrical signal by using USB-6008.

We used a MUT whose central frequency is 12 MHz. The size of the transducer is 3 mm × 1 mm × 0.9 mm. High-frequency multichannel ultrasonic research platform Vantage 64 LE (Verasonics, Kirkland, WA) was applied to emit ultrasound signals. The sample rate was set at 48 MHz. The pulse repetition frequency (PRF) was set at 500 Hz. In order to control the amount of data, every time we emitted ultrasound signals, we emitted signals at the PRF of 500 Hz for 0.4 s and spent 0.6 s storing data and displaying radio frequency (RF) signals in real time. So, each frame contained 200 RF signals. These RF signals were processed into one ultrasound intensity result of a single frame. In phantom experiments, the acquisition time of RF signals were the same as that of the light signal. For in vivo experiments, when emitting the ultrasound, the electrical signal and light signal were marked to highlight the period of brain activity which was associated with ultrasound signal. The ultrasound recording lasted including seizure and nonseizure which were shown by the ECoG signal.

### 2.2. Phantom Experiments

**Figure 1:**
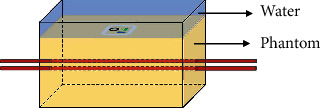
The design of phantom with two channels. The combination was placed above the two channels and close to the phantom surface. Water covers the combination for conducting ultrasound. The first channel was injected with 50% blood. The second channel was injected with different concentrations of blood.

In order to verify the effectiveness of MUT for detecting the CBV change in depth, we used a single-element MUT to detect the changeable concentration of blood in a phantom with wall-less channels. Although blood concentration is slightly different than CBV, it is directly related to the total hemoglobin (HbT). Given the fact that the brain volume is relative stable, the HbT change is usually considered CBV change. In our experiment, we perfuse different concentrations of blood in a tube and channels with fixed volume, which result to a change in HbT. We designed a gelatin phantom (100 mL water and 20 g powder) with wall-less channels whose diameters are 0.7 mm. The gap of the channels is 1 mm. The depth of the deeper channel is near 12 mm. The different concentrations of blood were pushed into the deeper channel. The different concentrations of water-lysed blood solution were prepared by the following steps: first, we used a syringe to slowly draw 10 mL 100% blood solution making sure that no bubbles were extracted. Next, we extracted 10 mL 0.9% saline solution in the same way. We mixed the two solutions to get 20 mL 50% water-lysed blood solution. Other concentrations of blood can be obtained in the same way. In Figure 1, on the surface of the phantom, we put the MUT directly above the channels covered with water. First, we filled the upper channel with water and the deeper channel with air. The transducer was rotated to the angle where the interfaces in the upper channel and deeper channel can be observed. Further, both channels were filled with water, and we can clearly distinguish the echo signals of the interfaces, which were between the channels and the gelatin phantom, on the RF signals. The distance between the two interface echoes of the deeper channel was 0.7 mm which was consistent with the channel diameter. It could be considered that the deeper channel was observed. The deeper channel was at a depth of 12 mm. So, it confirmed that the penetration depth of MUT could achieve at least 12 mm. Because of the need to process ultrasound signal properly, we had to ensure that the MUT was not perpendicular to the channels in the experiment. We needed to find the vertical angle first and then adjust the angle to make sure the MUT was not perpendicular to the channels. We adjusted the tilt of the transducer until the amplitude of the echo signal corresponding to the interface of the channel and gelatin phantom reaches the utmost. In this condition, the transmit direction of the transducer was considered perpendicular to the channel. Next, we tilted the transducer to achieve the angle which is not perpendicular to channels and make the amplitude become half of the biggest. In the case of the transducer tilted, we injected blood, collected data, and do further process. Next, the upper channel was injected by 50% concentration of blood. Different concentrations of blood, which were in the order of 50%, 75%, 100%, 75%, and 50%, were injected into the deeper channel with a fixed speed of 0.4 mL per minute. At the same time, we recorded the RF signals along the path of the transducer and channels. For each concentration of the blood solution, we injected for 2 minutes. Every time we switch the blood solution, we injected water into the channel to make sure that the new blood solution entering the channel is not affected by the blood solution remaining in the channel. All the RF data were processed offline by MATLAB software.

**Figure 2:**
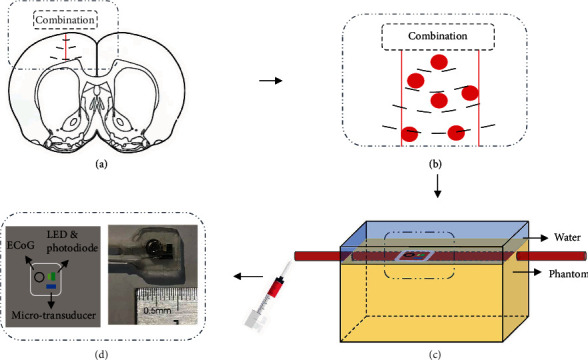
(a) The prototype of cerebral blood detection with combination. (b) The model of local cerebral blood detection with combination. (c) The design of phantom for realizing the model. The tube was attached to the phantom surface, and the combination was placed above the tube. The tube was injected with different concentrations of blood. (d) The schematic and the image of the combination.

After verifying the ultrasound effectiveness, the mimical epilepsy model was probed by the combination of the PPG device and MUT. In some cases, the blood concentration in the cortex changes in response to seizures. In Figure 2(a), the combination was used to detect the blood concentration change along the red line in the brain. The piece of change can be abstracted into the model in Figure 2(b). The blood concentration change under the combination can be detected. A phantom was designed that could be used to realize the model. A thick pipe was buried in the gelatin phantom, and the outer wall of the tube was close to the upper surface of the phantom in Figure 2(c). The outer diameter of the tube is 3 mm, and the inner diameter is 2 mm. This method was used to imitate the change of blood concentration in the cortex. Also, 50% and 100% blood was pushed into the tube in turn and detected by the combination which was covered with water vertically on the pipe. The position can be confirmed by superficial blood concentration changes which are provided by the PPG device and changes in the depth which are provided by the MUT. Due to the lack of conditions to imitate epileptic electrical signals in the phantom, only the optical part and the ultrasound part of the combination, which are shown in Figure 2(d), were used in the phantom experiment.

### 2.3. In Vivo Experiments

**Figure 3:**
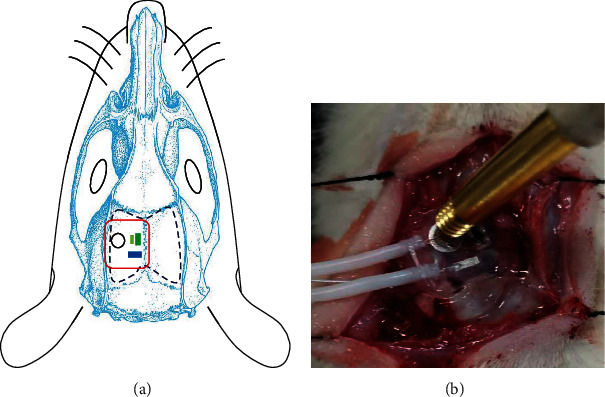
(a) The schematic of in vivo experiment. (b) The image of in vivo experiment setup.

The adult male Sprague-Dawley rats (250 g~300 g) were injected with urethan (0.3 g/mL). We fixed the head of rats in a stereotaxic apparatus. The skins over the head were sliced open. We drilled a cranial window on the rats' head. The area which the window covers was 10 mm × 10 mm. It is large enough for us to place the combination of PPG device, ECoG, and MUT on it. The necessity to open the window is to avoid the ultrasound attenuation when propagating into the brain. We kept dura, which has little effect on ultrasound signals, to prevent inflammation. We injected 4-aminopyridine (4-AP; Sigma-Aldrich) solution (25 mmol/L) with a capillary glass tube into the rats' brain. The epilepsy was induced by 4-AP. 4-AP is a K+-channel blocker that induces ictal-like events in the rodent neocortex and hippocampus. 4-AP is a highly reproducible model of acute focal seizures, whose electrographic patterns mimic the low voltage fast (LVF) activity seizure pattern found in human epilepsy [28–30]. The combination was put on the cortex to record signals in Figure 3.

### 2.4. Data Processing

Ultrasound RF signals were processed by the following steps: for each frame, a sequence of RF signals was emitted with 500 Hz PRF. We collected RF signals *s*(*z*, *t*), which contained the target area, and converted it into in-phase/quadrature (IQ) signals IQ(*z*, *t*) where *z* is the depth inside the phantom and *t* is the slow time. IQ signals are demodulated from a real signal to a complex signal with Equation (1) and filtered by a numerical Butterworth low-pass filter of fifth order with a cutoff frequency of *F*_c_.

(1) $$\begin{array}{c} \text{IQ}\left(z,t\right)=s\left(z,t\right)\times e^{-i\times 2\pi \times F_{\text{c}}\times z}. \end{array}$$

*F*_c_ is the central frequency of the transducer. *s*(*z*, *t*) could be the temporal signal of one sample point in depth and be decomposed into wall signal *s*_w_(*z*, *t*) and blood signal *s*_B_(*z*, *t*). In order to filter out the wall signal, we filtered the IQ signals in each frame along the slow-time dimension with a numerical Butterworth high-pass filter of fifth order in Equation (2)

(2) $$\begin{array}{c} \bar{s}\left(z,t\right)={\text{HPfilter}}_{t}\left(\text{IQ}\left(z,t\right)\right). \end{array}$$

The fixed cutoff frequency of the filter depended on blood flow rate. In phantom experiments, we set the cutoff frequency to 10 Hz. It is low enough to filter out phantom signal which is the equivalent of a human tissue signal. For in vivo experiments, we set the cutoff frequency to 60 Hz, because of the noise caused by tissue movement. After the IQ signals were filtered by a wall filter, data were processed by power Doppler methods with Equation (3) and Equation (4).

(3) $$\begin{array}{c} s_{\text{B}}\left(z,t\right)=\left|\bar{s}\left(z,t\right)\right|. \end{array}$$

(4) $$\begin{array}{c} I\left(z\right)=\frac{1}{N}\overset{N}{\underset{i=1}{\sum }}{s_{\text{B}}}^{2}\left(z,t_{i}\right). \end{array}$$

We calculated the sum of the square of blood signals along the slow-time dimension in a frame so that the result *I*(*z*) could describe the intensity of blood for each depth at that frame. Combining the results of many frames, we can show the change of blood concentration over time in the target area.

In phantom experiments, the injection procedure was recorded for calculating the correlation coefficient with US signal. For in vivo experiments, we smoothed the raw ECoG signals and light signals to apply into subsequent processing. We calculated the correlation coefficient between ultrasound results and task pattern with Equation (5)

(5) $$\begin{array}{c} r=\frac{\sum_{i=1}^{N_{t}}\left(s_{\text{B}}\left(t_{i}\right)-{\hat{s}}_{\text{B}}\right)\left(A\left(t_{i}\right)-\hat{A}\right)}{\sqrt{\sum_{i=1}^{N_{t}}{\left(s_{\text{B}}\left(t_{i}\right)-{\overset{\wedge }{s}}_{\text{B}}\right)}^{2}}\sqrt{\sum_{i=1}^{N_{t}}{\left(\text{A}\left(t_{i}\right)-\overset{\wedge }{A}\right)}^{2}}}. \end{array}$$

where *r* is the Pearson product-moment correlation coefficient, *s*_B_(*t*) is the blood signal for some depth, *A*(*t*) is the task pattern, and $\hat{\;}$ designates the mean value of a variable. For the phantom experiment, *A*(*t*) is the injection procedure and light signal. And for the in vivo experiment, *A*(*t*) is the envelope of ECoG and light signal whose raw signals were smoothed. It can be used to decide the place where seizures happen. The high correlation coefficient can be considered the right place.

## 3. Results

### 3.1. Phantom Experiment Result

**Figure 4:**
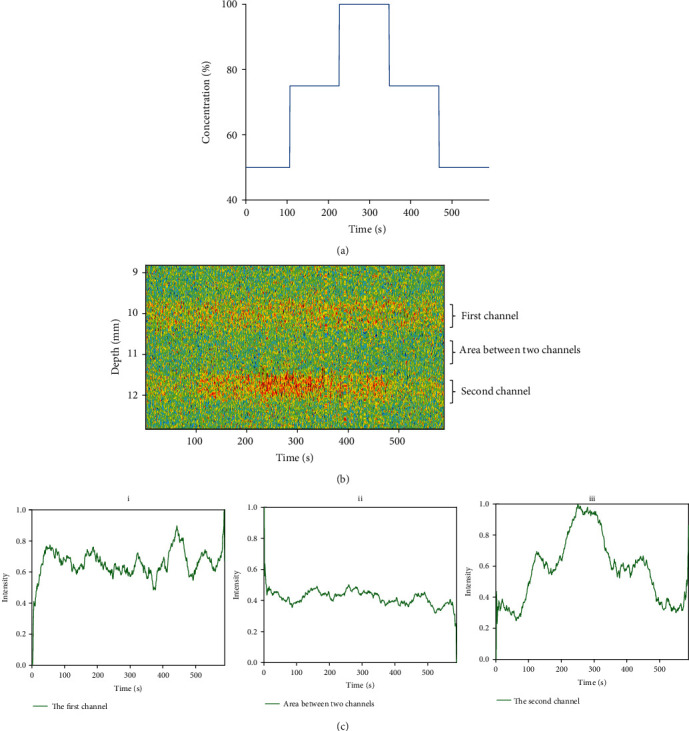
(a) The injection procedure in the deeper channel. (b) The result of ultrasound intensity ranged from 9 mm to 13 mm away from phantom surface. (c) The normalized ultrasound intensity in different areas: (i) the first channel, (ii) area between two channels, and (iii) the second channel.

**Figure 5:**
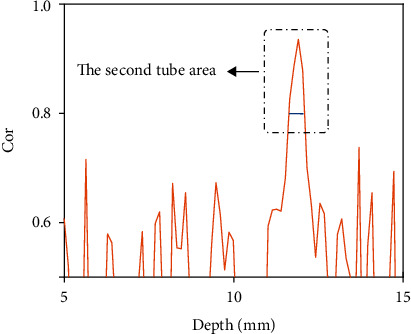
The Pearson product-moment correlation coefficient between the injection procedure and normalized ultrasound intensity from 5 mm to 15 mm away from surface in the phantom.

In the first phantom experiments, the blood concentration varied in the order of 50%, 75%, 100%, 75%, and 50% in the deeper channel. The signals whose depth were 20 mm away from the surface of phantom were recorded and processed. In Figure 4(a), the injection procedure of different blood concentrations was used for calculating the correlation coefficient.  Figure 4(b) showed the result of ultrasound. The channel's position was accurately located near 12 mm. The process of the change of blood concentration in the channel can be clearly observed. In the first channel, the blood flowed in a steady state. And in the second channel, the blood flowed with the change of concentrations. The normalized ultrasound intensity in different regions is depicted, respectively, in Figure 4(c). The situation in the second channel was most similar with the task procedure. It means that the change of 25% concentrations could be distinguished by the MUT. The correlation coefficients of ultrasound intensity in different depths ranging from 5 mm to 15 mm in the phantom were obtained, respectively, with the task procedure. In Figure 5, the Pearson product-moment correlation coefficient of second channel depth was higher than 0.8. The range of depth was outstanding and can be considered to correspond with the actual location. And the width of 0.6 mm was close to the actual channel diameter.

**Figure 6:**
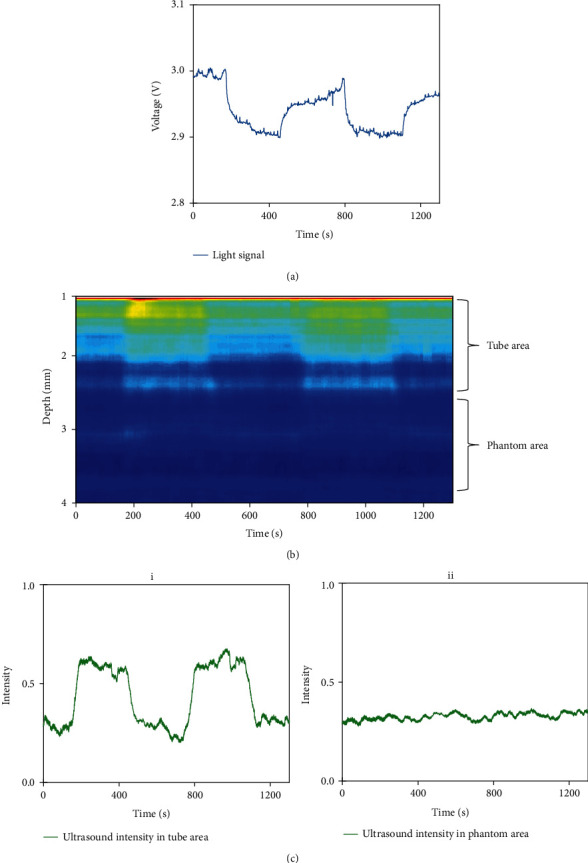
(a) The light signal changes with the injection procedure including 50% and 100% blood. (b) The ultrasound intensity from 1 mm to 4 mm away from the phantom surface. (c) The ultrasound intensity in different areas: (i) tube area and (ii) phantom area.

It confirmed that the ability to observe the blood concentration with the MUT can be devoted into a seizure-detecting application. Further, the combination needs to be verified if it works in the imitation of the epilepsy model.  Figure 6 shows the result in the second phantom experiments. In Figure 6(a), the light signal, which reflected the blood concentration on the surface of the phantom, declined at the high concentration of blood and rose at the low concentration. This is due to the absorption of light by blood. The higher blood concentration was accompanied by the weaker reflected light. This had a good correspondence with real fact.  Figure 6(b) only shows the ultrasound intensity from 1 mm to 4 mm away from transducer surface because 0 mm to 1 mm was covered by the blind zone of the transducer. The detected depth of changeable blood concentration reached 2.5 mm away from the surface. Considering the thickness of the tube outer wall (0.5 mm), the depth matched the inner diameter of the tube. Similarly, we observed the ultrasound intensity in the tube area and the phantom area in Figure 6(c). The inner part of the tube corresponded well to the light signal. The absolute values of correlation coefficients were 0.8 and 0.3, respectively. The result in the tube matched light signal precisely. This supplemented the depth information. It confirmed that the combination can be applied to the detection and location of blood concentration changes in the brain.

### 3.2. Animal Experiment Result

**Figure 7:**
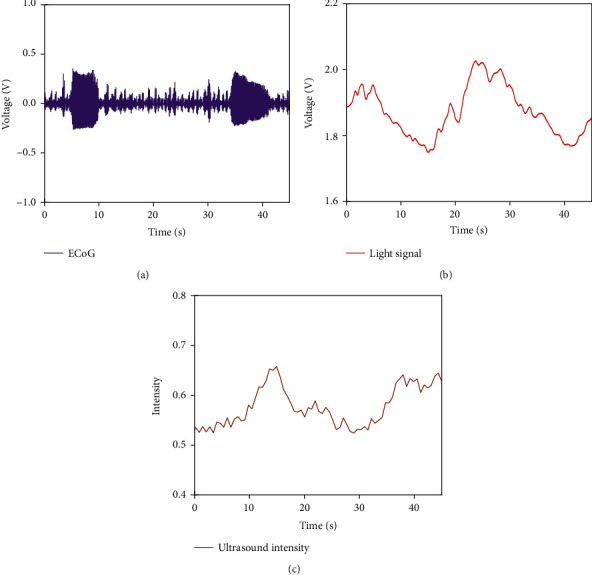
The in vivo experiment result: (a) the ECoG signal; (b) the light signal; (c) the ultrasound intensity.

The same experiment setup was used in vivo. We tried to find patterns of intracranial blood concentration during seizures. It can realize the epilepsy localization in the brain tissue by detecting blood concentration changes. In Figure 7, the electrical signal shows that there was a storm that happened on the brain surface. It means that at the time of 5 s~10 s and 35 s~40 s, seizure occurred at a range of 10 mm × 10 mm centered on the electrode position. The ECoG signal was treated as a task reference. The recorded voltage of the light signal descended a little bit later after the seizure times. So, the blood concentration on the surface of the brain rose up. It can be confirmed that the position of the seizure was near the PPG device, and there were blood concentration changes within 1 mm below the cortex. Further, the same case which the ultrasound detected also existed from 0.5 mm to 1.5 mm below the cortex; it had a good correspondence with the ECoG signal, and the change of blood concentrations is close to 25%. This indicated that the specific location of the seizure is 0.5 mm to 1.5 mm below the PPG device. The fact verified that it is feasible to realize the joint localization of epilepsy by using MUT, PPG device, and ECoG.

## 4. Discussion

In the first phantom experiments, the ability of the MUT to detect the change of blood concentration in depth was verified. The penetration depth of MUT could achieve at least 12 mm. The change of blood concentration in the second channel corresponded well to the task procedure. The correlation coefficient in the second channel was higher than 0.8. The results of the correlation coefficient can well highlight the target position and distinguish it from other depths. The design that a certain concentration blood filled in the upper layer was to demonstrate that changes in the deeper channel can still be detected by the MUT. The central frequency of the MUT is 12 MHz. It could provide the enough resolution of detection. In the first phantom experiments, the smallest channel that it can detect was 0.7 mm and the gap of the two channels was less than 1 mm. If channels with a smaller diameter can be made, the author believes that the method could also detect the blood concentration change by the MUT.

The second phantom experiments verified the feasibility of detection with combined PPG device and MUT. Light could reflect the blood concentration change on the surface. The change of blood concentrations cannot be detected when the PPG device was more than 1 mm away from the phantom surface. So, the PPG device can detect the blood concentration change within 1 mm below the device position but cannot locate the specific depth. The results showed that the ultrasound intensity in the tube had the same trend with the light signal and gave the exact depth where blood concentration changed. With the assistance of a MUT, the exact depth of blood concentration change can be determined.

For in vivo experiments, the electrical signal accurately expressed the moment of seizure. Due to the slow change of blood, the changes of light signal and ultrasound intensity were slightly delayed, but the overall trend was consistent with the electrical signal. The results of the in vivo experiment confirmed the effectiveness of this combination.

In the ECoG method, the ECoG electrodes were separated with a gap of 1 mm, because the close distance between the two electrodes cannot distinguish the accurate position. Besides, it also cannot provide the depth of seizure. The results of phantom experiments showed that our method relied on the PPG device, which could be placed less than 1 mm, to assist ECoG electrodes to improve the spatial resolution. It can define a more precise surface area according to its size. With the support of the MUT, we can map the seizure depth underneath the MUT.

It proved that the combination can detect lesions not only on the surface but also in the deep. It can also expand the individual combination into a net structure like the ECoG method. In clinical setting, the epileptogenic foci were located through EEG, brain imaging, or other methods. Usually, the patients were implanted ECoG arrays and were performed cortical potential recording, until seizures were captured. The epileptogenic foci were located according to the cortical potential recording. Finally, according to the localization of epileptogenic foci, a second operation was performed to remove the epileptogenic foci. Our new method upgrades ECoG in the same way as ECoG without changing clinical habits and procedures. So, this is a novel method and application which can easily be used in the clinical setting. This could achieve a real-time detection for weeks or months so that it could cover the whole procedure of the seizure events.

In the results of in vivo experiments, typical seizure results showed that the ECoG signal, light signal, and ultrasound intensity had a good corresponding relationship. ECoG and PPG devices could be used to confirm the specific seizure location, and the depth of selected ultrasound intensity which has with a good corresponding relationship could be used to confirm the location of epileptic lesions inside the brain. Currently, our method has been applied to rat brains to assess the performance, and will be further tested in larger animals, such as rabbits and dogs. And we are looking forward to applying this method in the clinical setting in the future or devoting this combination in other research about epilepsy and other brain diseases.

## 5. Conclusions

In the application of detection of neocortical cryptogenic epilepsy, the ECoG method has some limitations. This paper proposed a new combination based on the ECoG method, which combines the PPG device and MUT, to improve the detection ability of superficial blood concentration change and provide the depth information which is lacking in the ECoG method. Phantom experiments and in vivo experiments were designed to verify the combination. The first experiments designed a phantom with two channels at different depths, and the second experiments imitated that the blood concentration changed in the cerebral cortex. The results showed that the MUT could still find the change of blood concentration at the second channel even with 50% concentration of blood in the first channel and could distinguish the 25% concentration changes. The correlation coefficient of the second channel was as high as 0.8, which distinguished it from other depths and realized the positioning function. In the second experiment results, the superficial blood concentration change detected by the PPG device and the deep change detected by the MUT have a good corresponding relationship. Moreover, the range of blood concentration change detected by ultrasound was consistent with the actual tube diameter, which supplemented the specific depth information in detection. Finally, the combination was verified for in vivo experiments. The results of in vivo experiments showed that the light signal corresponded to the ECoG signal, indicating that there was a blood concentration change under the position of the device. The ultrasound intensity below the cortex of 0.5 mm to 1.5 mm also has a good corresponding relationship with the ECoG signal. ECoG, PPG device, and MUT work together to identify the moment, location, and depth of the seizure.

## Data Availability

The data used to support the findings of this study are available from the corresponding author upon request.
